# Organoids: new frontiers in tumor immune microenvironment research

**DOI:** 10.3389/fimmu.2024.1422031

**Published:** 2024-07-29

**Authors:** Yujia Yang, Jinlei Cui, Yajie Kong, Yu Hou, Cuiqing Ma

**Affiliations:** ^1^ Department of Immunology, Key Laboratory of Immune Mechanism and Intervention on Serious Disease in Hebei Province, Hebei Medical University, Immunology Department of Hebei Medical University, Shijiazhuang, China; ^2^ Hebei Medical University-National University of Ireland Galway Stem Cell Research Center, Hebei Medical University, Shijiazhuang, Hebei, China

**Keywords:** organoid, immunotherapy, tumor microenvironment, precision medicine, cancer

## Abstract

The tumor microenvironment (TME) contains cells that regulate medication response and cancer growth in a major way. Tumor immunology research has been rejuvenated and cancer treatment has been changed by immunotherapy, a rapidly developing therapeutic approach. The growth patterns of tumor cells *in vivo* and the heterogeneity, complexity, and individuality of tumors produced from patients are not reflected in traditional two-dimensional tumor cell profiles. On the other hand, an *in vitro* three-dimensional (3D) model called the organoid model is gaining popularity. It can replicate the physiological and pathological properties of the original tissues *in vivo*. Tumor cells are the source of immune organoids. The TME characteristics can be preserved while preserving the variety of tumors by cultivating epithelial tumor cells with various stromal and immunological components. In addition to having genetic and physical similarities to human diseases and the ability to partially reconstruct the complex structure of tumors, these models are now widely used in research fields including cancer, developmental biology, regenerative mechanisms, drug development, disease modeling, and organ transplantation. This study reviews the function of organoids in immunotherapy and the tumor immune milieu. We also discuss current developments and suggest translational uses of tumor organoids in immuno-oncology research, immunotherapy modeling, and precision medicine.

## Graphical Abstract

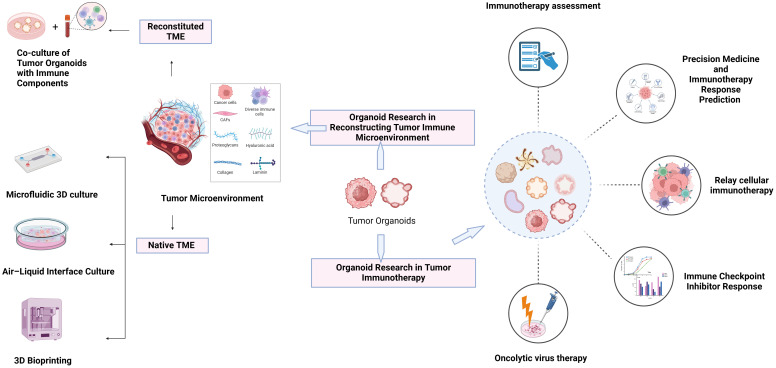

## Introduction

**Figure 1:**
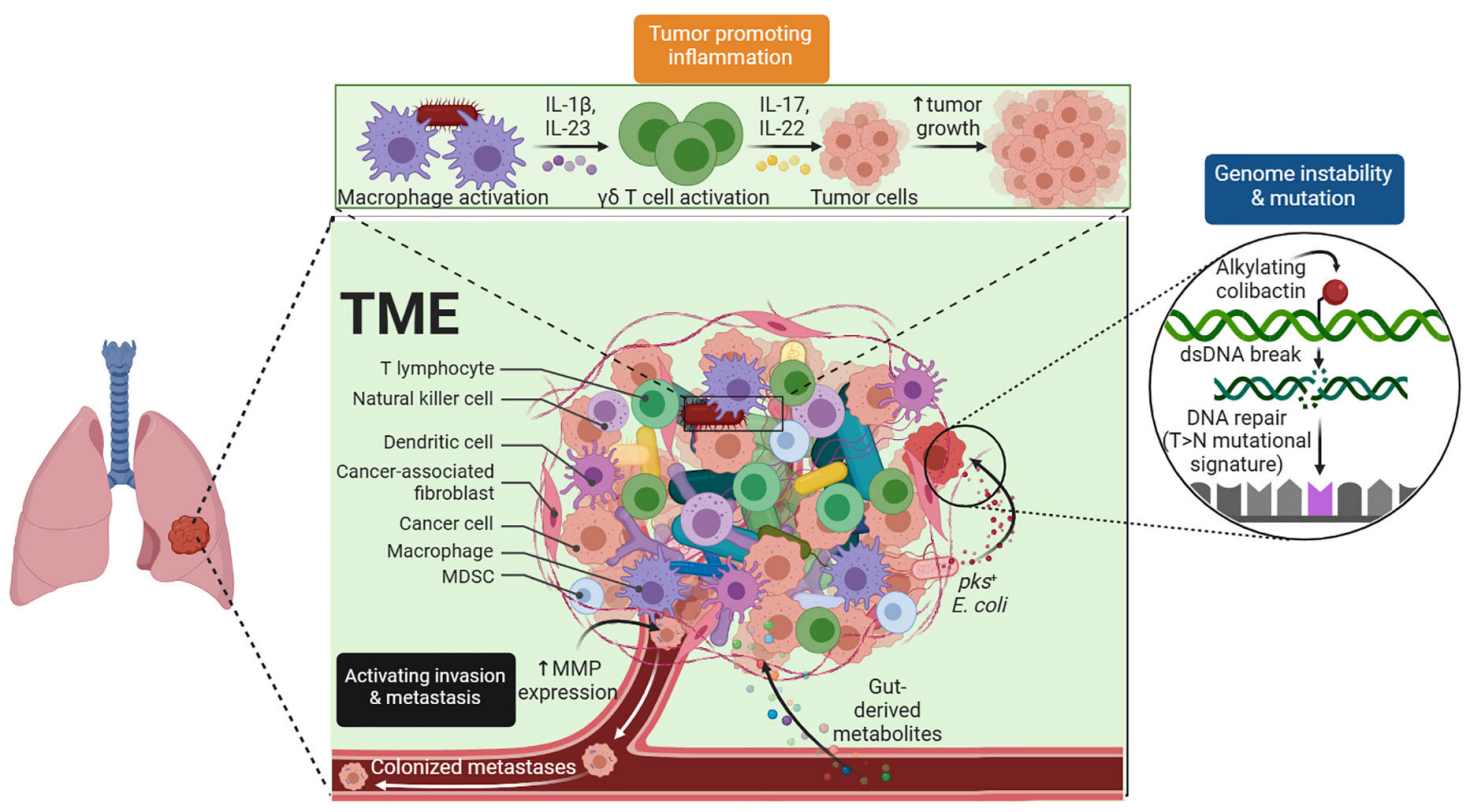
Tumor Microenvironment. The complex TME, which includes tumor cells, blood vessels, immune cells, and stromal components, is critical for tumor development and treatment response.

The TME is an intricate system. It comprises not only tumor cells but also a variety of other cell types, such as blood vessels, activated fibroblasts, invading immune cells, and the extracellular matrix, that are involved in the growth of tumors (**Figure 1**). Understanding carcinogenesis, tumor growth, and tumor metastasis depends on understanding the TME, which is regulated by a range of cellular, hormonal, and inflammatory responses. As a result, this topic has long been a goal of cancer research and plays a significant role in the prevention, diagnosis, and treatment of cancers. Research has demonstrated that the TME can affect the course of tumors and how well immunotherapy drugs work (1).

Coculturing two-dimensional tumor cells with foreign immune cells has become a standard technique for preclinical trials of pertinent immunotherapeutic medicines as well as fundamental tumor immunology research. It has a large variety of assays and well-established evaluation methodologies in addition to allowing straightforward gene editing and medication intervention. Tumor cell profiling, however, does not capture the *in vivo* growth pattern of tumor cells. Traditional two-dimensional tumor cell cultures have significant limitations; they fail to accurately represent the growth patterns, heterogeneity, complexity, and individuality of patient-derived tumors observed *in vivo*. The 2D culture model cannot accurately reflect the three-dimensional growth patterns of tumor cells or the spatial and temporal distribution characteristics and interaction modes between tumor and immune cells *in vivo*. Additionally, 2D cultures cannot realistically reproduce the tumor microenvironment, including interactions with stromal and immune cells, often leading to a loss of cellular heterogeneity. Long-term passaging culture may result in genetic mutations and phenotypic variance in additional immune cells, as they are unable to replicate the temporal and spatial properties and interaction patterns of the TME (2).

Inflammatory and immunodeficient conditions have historically been linked to cancer susceptibility, indicating the central role of immunity in carcinogenesis. Immunotherapy-based revolutionary methods for cancer treatment have recently been shown to be efficacious. Both medicines and cells are known to have immunomodulatory effects. These immunotherapies have the ability to locally modify the tumor’s immunological milieu while also methodically enhancing the body’s immunity. Tumor antigen-targeted monoclonal antibodies, vaccines, therapies targeting pattern recognition receptors (PRRs), and other nonspecific small molecules, such as interleukins (ILs), interferons, and colony-stimulating factors, are among the immunotherapies that have been employed in clinical settings (3, 4). Adoptive cell therapy (ACT) and immune checkpoint inhibitors (ICIs) are two examples of immunotherapy that have altered the conventional paradigm for treating tumors (5–8). However, the number of patients available for participation in these studies is still quite limited. The primary cause is that patients can be inherently resistant to immunotherapy due to a variety of factors, including genetic mutations, the presence of certain biomarkers, variations in the tumor microenvironment, and differences in immune system functioning. This inherent resistance is difficult to demonstrate using conventional immunological research techniques, which do not adequately capture the complex interactions between tumor cells. To conduct basic and clinical translational research on tumor immunity, it is therefore vital to design new preclinical models that accurately replicate the human tumor immune milieu (9, 10).

Immune organoids are three-dimensional culture systems derived from immune cells or containing immune cell components. They are designed to mimic the structure and function of the immune system or its specific parts. Organoid culture systems have become important resources for researching the interactions between cancer cells and other elements of the tumor immunological microenvironment and for simulating the TME (11). For instance, polymorphonuclear myeloid-derived suppressor cells (PMN-MDSCs) have been demonstrated to increase tumor growth and limit the proliferation of cytotoxic T lymphocytes (CTLs), hence reducing the efficiency of checkpoint inhibition in murine and human-derived autologous organoid/immune cell cocultures (12). To mimic tumor-specific immune responses and prevent alloreactivity, autologous tumor-infiltrating lymphocytes (TILs) have been added to 3D organoid cultures obtained from patient-derived xenografts (PDXs) (13). Knowing how dendritic cells (DCs) and colorectal cancer (CRC) cells interact in the immunosuppressive TME may help researchers discover important pathways involved in tumor growth and dissemination for treatment (14). Organoid technologies for mimicking cancer have advanced significantly. One option is to use forward genetic techniques, which involve modifying induced pluripotent stem cells (iPSCs) or cells produced from wild-type tissues to have oncogenic or cancer-suppressive mutations (15, 16). However, the strong dispersion of patient-derived human tumor biopsies (PDOs) is now made possible via organoid techniques. The widespread use of 3D-PDO cultures has significantly influenced the field of *in vitro* cancer biology, resulting in the establishment of extensive tumor libraries that encompass the genetic and histological diversity of human malignancies. This paper reviews the function of organoids in immunotherapy and the tumor immune microenvironment.

## Organoid research in reconstructing the tumor immune microenvironment

**Figure 2:**
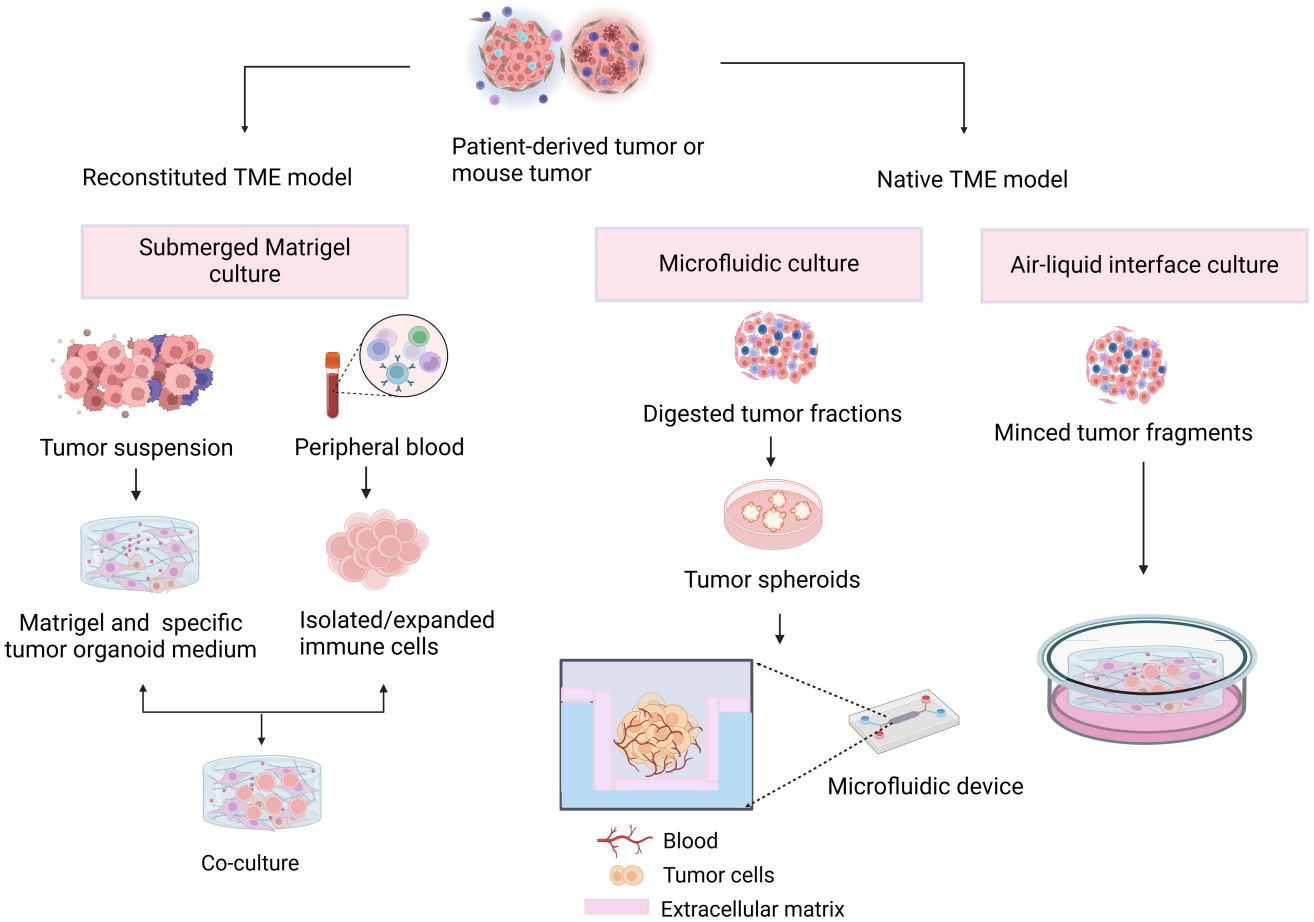
Different approaches to tumor immune microenvironment generation. In the reconstructed model, organoids containing tumor cells are cocultured with exogenous immune cells. In the nonreconstructed model, preservation of the inherent immune environment of the tumor specimen by injection or implantation of a collagen gel containing tumor cells and immune components.

In organoids, the tumor immune microenvironment can be affected by two different methods (**Figure 2**). In reconstituted models, organoids containing exclusively tumor cells, often from physically and enzymatically separated tissues, are cultivated in extracellular matrix domes (e.g., Matrigel or BME-2) and submerged in tissue culture media. After being separated, exogenous immune cells—such as those from autologous peripheral blood or tumors—are cocultured with developed organoids. Without reconstitution, the intrinsic immunological milieu of tumor specimens is retained in holistic native TME models in addition to the tumor cells themselves. It is possible to combine collagen and tumor spheroids from digested tumor tissues for injection into microfluidic culture apparatuses. As an alternative, collagen gels are implanted in inner transwell dishes containing minced primary tissue pieces comprising both tumor cells and immunological components in air–liquid interface (ALI) culture. Because the top of the collagen gel is open to the air, cells can obtain enough oxygen to function. The effects of different culture models on the function and phenotype of various immune cell populations are shown in **Table 1**.

**Table 1:** Effects of different culture models on the function and phenotype of various immune cell populations.

Model	Description	Effects on T Cells	Effects on Macrophages	Effects on Dendritic Cells	Effects on NK Cells
Submerged matrix gel culture method	Embedding organoids in a matrix with exogenous immune cells.	a. Supports T cell infiltration and activation. b. Enhances T cell-tumor cell interactions. c. Promotes cytokine production.	a. Supports macrophage infiltration and polarization. b. Influences macrophage-tumor cell interactions.	a. Enhances DC-tumor cell interactions. b. Supports DC maturation and antigen presentation.	a. Promotes NK cell infiltration and cytotoxic activity. b. Enhances NK cell-tumor cell interactions.
Microfluidic 3D cell co-culture method	Utilizes microchannels for precise manipulation of cell positioning and nutrient flow.	a. Allows detailed analysis of T cell migration and infiltration. b. Enables study of T cell dynamics under flow conditions.	a. Enables study of macrophage migration and polarization under controlled conditions. b. Supports detailed phenotype analysis.	a. Allows precise control of DC positioning and interactions. b. Facilitates real-time monitoring of DC behavior.	a. Enables detailed study of NK cell migration and cytotoxicity. b. Supports phenotype analysis under physiological flow conditions.
Air–Liquid interface (ALI) culture method	Exposes organoids to air on the upper surface and provides nutrients from the liquid medium below.	a. Maintains T cell-tumor interactions over long-term. b. Supports T cell activation and memory formation. c. Allows for studies of immune infiltration.	a. Maintains macrophage-tumor interactions. b. Supports macrophage polarization and function. c. Preserves macrophage diversity within TME.	a. Supports long-term DC-tumor interactions. b. Enhances antigen presentation and immune activation. c. Preserves DC diversity and function.	a. Maintains NK cell-tumor interactions. b. Supports NK cell cytotoxicity and function. c. Preserves complex immune cell interactions within TME.

3D bioprinting is also an advanced biocultural technology that uses layers of biomaterials to construct complex tissue and organ structures.

### Coculture of tumor organoids with immune components

Organoid technology has progressed recently, offering more precise models to examine medication response, the TME, and tumor growth, revolutionizing research on cancer. In examining interactions between cells and between cancer cells within the TME, organoid models have proven to be especially useful (17). To investigate tumor-immune interactions and prospective therapeutic approaches for cancer treatment, coculture systems that include immune components and cancer organoids have become attractive.

**Figure 3:**
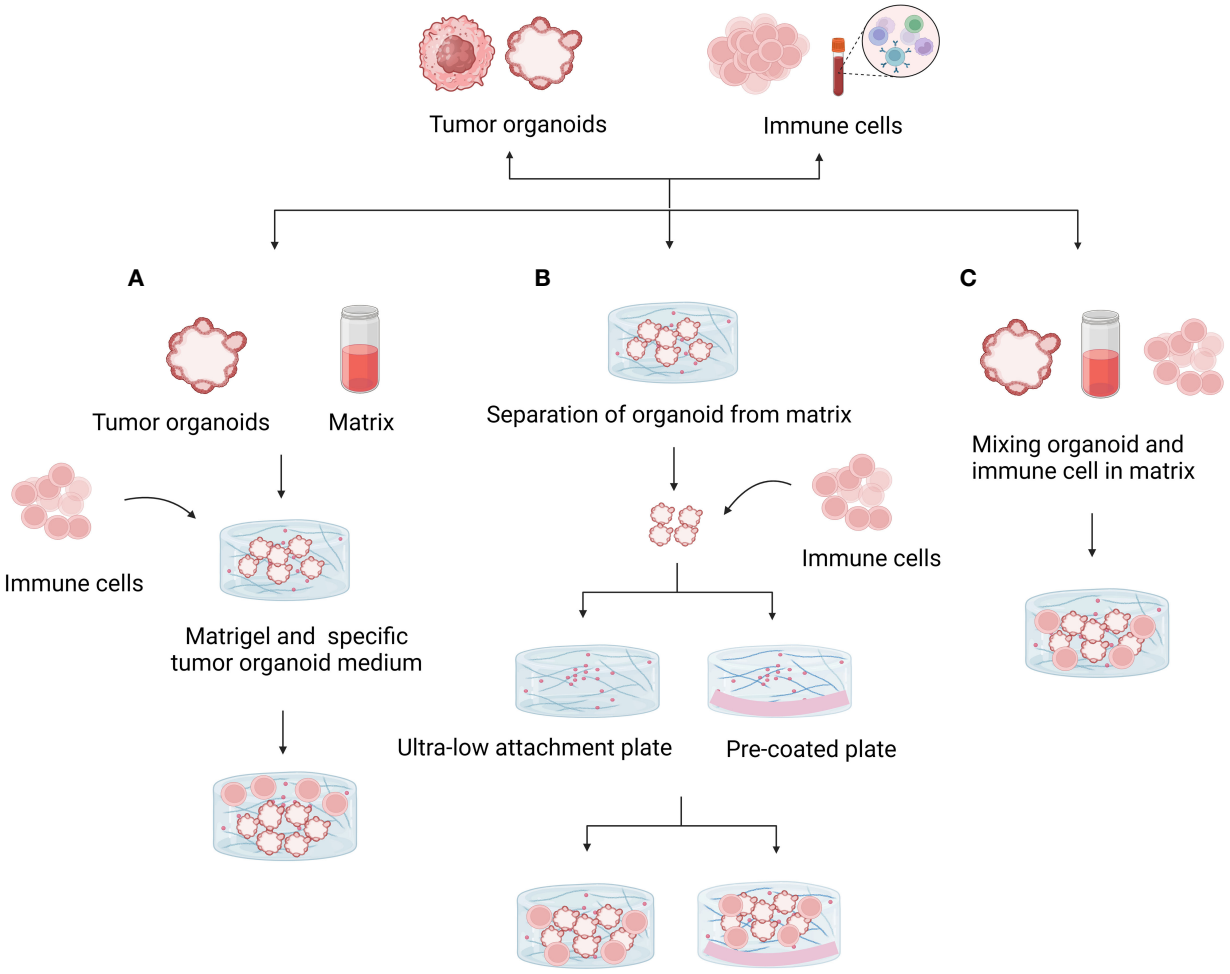
Methods for coculturing tumor organoids and immune cells. **(A)** Mixing the organoid and matrix and adding exogenous immune cells to the medium promoted indirect interactions. Matrix dilution facilitates immune cell infiltration and direct contact with tumor cells. **(B)** Isolated organoids were mixed with suspended immune cells to study direct interactions. The use of ultralow adhesion culture plates or plates precoated with substrate prevents organ adhesion and death. **(C)** Mixing organoids and immune cells directly in the matrix and culturing them in culture plates is a useful model for studying interactions.

By adding different elements, such as immune cells, cancer-associated fibroblasts, tumor vasculature, and other biological or chemical components, these organoid models seek to replicate the TME. Exogenous immune cells based on Matrigel deep culture must be added to immune organoid culture to model TMEs. The first method involves mixing organoids with the matrix and adding exogenous immune cells to the medium, which promotes indirect interactions. The second method is to first establish tumor organoids and, after a period of cultivation, dissociate the tumor organoids into single cells. At the same time, add lymphocytes extracted from peripheral blood to the dissociated tumor organoids, resuspend them in Matrigel, and re-embed them. The third method is to directly mix organoids and immune cells in the matrix and culture them in a dish (**Figure 3**). Additionally, the co-culture conditions need to be adjusted based on different tumor tissues and the added immune components (18). **Table 2** provides a detailed comparison of the three methods for establishing tumor organoids with immune components, highlighting their respective advantages, disadvantages, and effectiveness in immune cell reconstruction.

**Table 2:** Comparison of methods for establishing tumor organoids with immune components: advantages, disadvantages, and immune cell reconstruction.

Method	Advantages	Disadvantages	Comparison in Immune Cell Reconstruction
Mixing organoids with matrix and adding exogenous immune cells to the medium	a. Promotes indirect interactions between immune cells and tumor cells. b. Facilitates immune cell infiltration and closer contact with tumor cells.	a. Complex interaction dynamics are difficult to dissect and analyze. b. Variability in immune cell penetration can lead to inconsistent results.	Offers a model for studying paracrine signaling and other indirect interactions, but with variable immune cell infiltration.
Dissociating established tumor organoids and adding lymphocytes extracted from peripheral blood, then re-embedding in Matrigel	a. Allows for direct study of immune cell-tumor cell interactions. b. Prevents organoid adhesion and subsequent cell death.	a. Dissociation and re-embedding may damage cells. b. Multi-step process increases complexity and cost.	Provides clear insights into contact-dependent mechanisms but may lack the physiological relevance of the tumor microenvironment.
Directly mixing organoids and immune cells in the matrix	a. Provides a comprehensive model for both direct and indirect interactions. b. Embedding immune cells within the matrix enhances their integration and interaction with tumor cells.	a. The complexity of the matrix can make it difficult to isolate specific interactions or effects. b. Technically challenging to optimize for consistent results.	Offers a robust model for studying both direct and indirect interactions within a supportive matrix environment.

The success of targeted therapeutics has been predicted using autologous pancreatic cancer organoid/immune cell cocultures produced from mice and humans, underscoring the importance of including immune components in preclinical models (12). Further insight into immunosuppression in cancer has come from the addition of immune cells to organoid models. The incorporation of immune cells and stromal components into three-dimensional organoid models has made it possible to gain a more thorough understanding of the TME and its function in immunosuppression (19). The significance of immune cell contacts in the progression of cancer has been highlighted by the characterization of angiocrine crosstalk and the generation of an inflammatory TME using coculture models of hepatocellular carcinoma organoids with endothelial cells (20). Additionally, the creation of coculture techniques utilizing immune cells and patient-derived cholangiocarcinoma organoids has demonstrated potential in simulating anticancer immunity *in vitro* (21). The significance of these organoid-immune coculture models in furthering oncoimmunology research is highlighted by their ability to clarify tumor-specific immune interactions and direct customized immunotherapies (22). To summary, the incorporation of immune components into cancer organoid coculture models has yielded significant insights into immunosuppressive mechanisms, tumor-immune interactions, and possible therapeutic approaches. These models could increase our knowledge of the biology of cancer and direct the creation of tailored immunotherapies to improve patient outcomes (23).

### Microfluidic 3D culture

Microfluidic or 3D materials, which are more adaptable for imitating the TME, are placed into the channels and chambers of the microfluidic culture system (24). Controlling fluid flow and volume, as well as designing microchannels, can enhance the quality of organoids. It facilitates the mass manufacture of organoids and supports uniformity and controllability (25). Microfluidic chambers make up the microfluidic core. Every microfluidic chamber features a media conduit on both sides in addition to a center gel area. To create microspheres comprising tumor cells, immune cells, and stromal cells, fresh tumor tissue must first be chopped and beaten. Subsequently, the tumor microspheres are doped into a hydrogel made of collagen and injected into the center of the gel, strolling through the middle channel. Using a microfluidic device, Ahn et al. (26) created a bone-mimetic microenvironment to study the interactions between tumor cells and hydroxyapatite (HA) in a three-dimensional composite. The significance of building a three-dimensional TME for researching cell-cell interactions is emphasized by this work. The transition from planar to 3D cell culture on scaffolds, as well as the use of cell scaffolds in microfluidic devices, was covered by Castiaux et al. (27). Microfluidic platforms investigate a variety of scaffold types, including suspension culture, hydrogel scaffolds, paper-based scaffolds, and fiber-based scaffolds. Mulas et al. (28) presented a technique that uses hydrogel bead compartmentalization to encapsulate living cells in a 3D hydrogel matrix. This method highlights the versatility of 3D cell encapsulation in microfluidic devices by allowing retrieval for molecular and functional tests as well as monitoring of cellular dynamics. A quantitative image-based cell viability (QuantICV) assay was proposed by Ong et al. (29) for microfluidic 3D tissue culture applications. This assay method is a useful tool for drug testing applications because it overcomes the difficulties associated with conducting quantitative cell viability studies in microfluidic 3D tissue cultures. Specifically, the QuantICV assay employs advanced techniques such as high-throughput screening, which allows for the simultaneous automation of multiple assays to obtain precise and reliable data. It also utilizes fluorescent and luminescent dyes that bind to live or dead cells, enabling sensitive detection and accurate measurement of cell viability. Additionally, real-time monitoring through live-cell imaging systems provides dynamic data by tracking changes in cell viability over time. In summary, combining 3D cell culture methods with microfluidic technology is a viable strategy for examining cell behavior in a setting that is more physiologically appropriate.

### Air–liquid interface culture

The technique of physically splitting tumors into tissue fragments and then growing them in a cell culture chamber coated with collagen gel is known as ALI culture. This paradigm allows tumor cells to proliferate properly and create tumors that mimic the original tumor, preserving the pathological features and genetic alterations of the original tumor, in contrast to the limitations of the coculture model in analyzing the TME. The top of the gel is open to the outside, and a permeable cell culture chamber allows the medium from the outer dish to permeate into the inner dish, forming an air-liquid interface that helps the cells obtain enough oxygen. This strategy maintains the intricate histological structure of the tumor parenchyma and mesenchyme in the TME, together with a range of naturally occurring immune cells, including functional TILs, and encourages tumor growth in its native state (30).

Initially, ALI organoids established from normal tissues of different sites, including the small intestine, colon, stomach, and pancreas, included both epithelial and stromal components. Subsequently, the ALI organoid method has been used to culture organoids from human biopsy tissues, such as melanoma, renal cell carcinoma, and non-small cell lung cancer (NSCLC), as well as syngeneic mouse tumor organoids with immune reactivity (30). ALI PDOs not only retain the genetic alterations of the original tumor but also preserve the complex cellular composition and structure of the TME. Both the tumor parenchyma and stroma are maintained, including fibroblasts and various endogenous infiltrating immune cell populations.

Organoid air-liquid interface cultures are being increasingly used in a variety of scientific fields. Ao et al. (31) created a microfluidic technology that includes perforable culture chambers and an air-liquid interface to simulate the effects of cannabis exposure during pregnancy on the development of the human brain. This platform enables the creation of many organoids without fusing or streamline fabrication processes. To better understand the pathophysiology of COVID-19, Lamers et al. (32) proposed a human 2D air-liquid interface culture system for studying SARS-CoV-2 infection of human alveolar type II-like cells. Sette et al. (33) investigated cystic fibrosis *in vitro* using air-liquid interface cultures and organoid models made from patient-derived nasal epithelial stem cells, and demonstrated that these models are appropriate for pharmacological testing of cystic fibrosis transmembrane conductance regulator (CFTR). The significance of three-dimensional culture for kidney organoids to better replicate *in vivo* conditions and adjust the extracellular matrix composition was emphasized by Geuens et al. (34). Using an air-liquid interface method, Cham et al. (35) reported the creation of a highly biomimetic organoid culture system that resembles native immature testis tissue, including the vasculature. Additionally, Boecking et al. (36) created human airway epithelial organoids with apical membranes oriented externally. These organoids and air-liquid interface cultures displayed identical gene expression profiles associated with bronchial development and ion transport. A different technique for growing airway organoids formed from nasal brushing from a 2D air-liquid interface culture was published by Amatngalim et al. (37), which made it possible to consistently detect CFTR modulator responses in patients with cystic fibrosis. Overall, research and treatment development can benefit greatly from the literature’s depiction of the adaptability and usefulness of organoid air-liquid interface culture in simulating a range of biological processes, illnesses, and pharmacological reactions.

### 3D bioprinting

The structuring of droplets on a surface coated with cells is the basis for the 3D tissue reconstruction technique known as bioprinting. A matrix of extracellular and tumor cells is present in these droplets. This concept has the advantage that the tissue can be fully rebuilt and that the droplets can be mechanically or chemically treated to acquire the appropriate tissue characteristics. As a result, bioprinting enables extremely accurate tissue and cell implantation. To enhance the printing of brain tumor organoids, Clark et al. (38) developed a bioprinting technique that includes monitoring the behavior of the supporting fluid. Frankowski et al. (39) addressed problems pertaining to the establishment of biological connections and the comprehension of tissue structure and discussed the use of 3D bioprinting to create human tissues and organoids for preclinical pharmacological investigations. The state of the art in organoid bioprinting was discussed by Cabral et al. (40), along with its possible applications in regenerative medicine, drug discovery, and tissue engineering.

Ultimately, Shrestha et al. (41) developed a novel microarray 3D bioprinting technique intended to generate human liver organoids in a repeatable way, resolving the technical drawbacks of conventional culture techniques and enabling a thorough assessment of the cumulative effects of hepatotoxicity. The body of research on organoid 3D bioprinting indicates notable advancements in printing technology, biolinking, and applications across a range of biological domains.

## Organoid research in tumor immunotherapy

**Figure 4:**
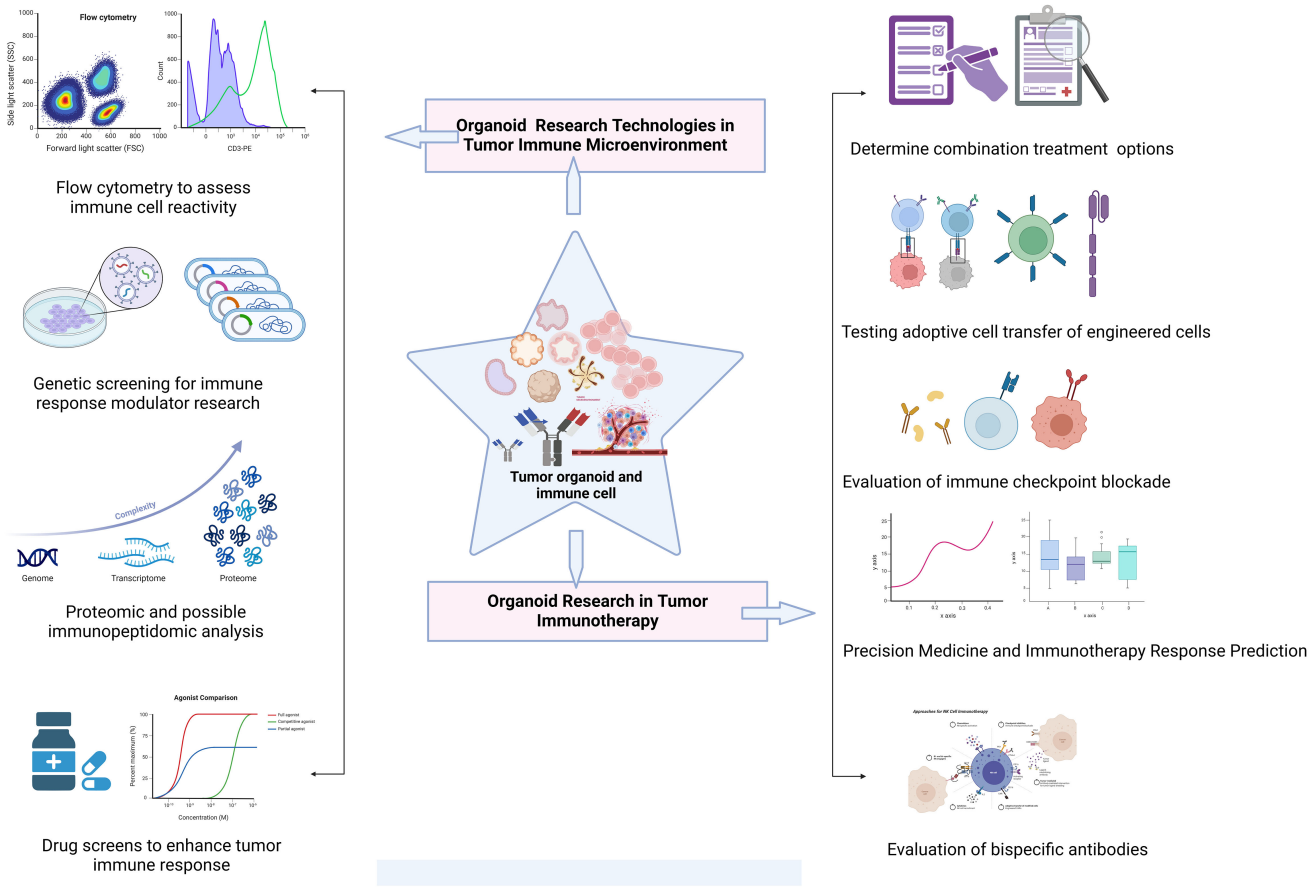
Exploration of common technologies and applications of organoid systems in tumor immunotherapy research. Tumor immunotherapy research using organoid systems expand the frontiers of personalized therapy and provides an important experimental platform for exploring novel immunotherapies.

The microenvironment around tumor cells functions as a unit. The microenvironment engages in interactions and coevolution with tumor cells, exerting a significant influence on various aspects of carcinogenesis and development. As a result, research on the interactions between tumors and their microenvironment provides theoretical groundwork for the development of novel therapeutic targets and novel strategies for tumor immunotherapy and aids in our understanding of the biological behavior of tumors (**Figure 4**).

### Immunotherapy assessment

Numerous promising therapeutic outcomes of immunotherapy have been identified thus far due to intrinsic and acquired resistance, and it is evident that certain tumor tissues are immune checkpoint resistant. Organoids may be used as viable substitutes for *in vivo* techniques, enhancing the efficacy of already available immunotherapies and increasing the viability of novel strategies.

Preclinical testing of immunotherapy medications for lung cancer and other cancer types can be carried out by researchers by imitating immunotherapy responses using organoid culture platforms (42). Organoid models produced from patients have been especially useful in the evaluation of immunotherapies for melanoma (43). These models allow immune cells within the organoids or new tumor samples to be assessed, which offers important insights into how the immune system reacts to therapy. Furthermore, organoids have been effectively developed for the treatment of breast cancer and are used as *in vitro* models to assess immunological responses and medication sensitivity (44). Moreover, models of organoid culture have been created to facilitate the repetitive assessment of immune cell interactions and tests for killing *in vitro* (45). Organoid models have been used in the setting of lymphoma to replicate the death of cancer cells by T cells upon treatment with bispecific immunotherapies (46). Comparably, a tumor organoid platform that engages T cells has been created for pancreatic cancer immunotherapy, enabling monitoring of the immune profile and assessment of the effectiveness of the treatment (47). In conclusion, organoids produced from patients have proven to be useful instruments for assessing immunotherapies in different kinds of cancer.

### Immunotherapy of engineered cells

Adoptive cell immunotherapy typically involves isolating immune cells from tumor patients, expanding and functionally characterizing them *ex vivo*, and then reinfusing them into the patient. Through genetic engineering, T cells can be combined with chimeric antigen receptors (CARs), and engineered chimeric antigen receptor T (CAR-T) cells can recognize tumor antigens, thereby achieving the goal of directly killing tumor cells or stimulating the body’s immune response to eliminate tumor cells.

Innovative organoid-T-cell coculture systems that offer a physiologic depiction of the interactions within the TME have been developed by Hubrecht Organoid Technology (HUB) (48). These HUB organoids, derived from both healthy and sick tissues, provide an excellent and repeatable platform that replicates the intricate features of the original parental tissue, such as functional properties and molecular heterogeneity. By evaluating T-cell cytotoxicity against tumor organoids and viewing and quantifying T-cell-tumor interactions, HUB’s organoid-T-cell coculture method facilitates the evaluation of novel therapeutics. This platform has promise as a robust instrument for the advancement and verification of cancer immunotherapy, encompassing immune checkpoint inhibition and bispecific antibody administration. Furthermore, TILs and CAR-engineered T cells may be able to function in cancer organoid-T-cell culture systems, providing prospects for preclinical development and customized therapeutic approaches. By offering a customized method for researching immune cell types and adoptive therapy, these organoid models may increase the effectiveness of immunotherapy treatments (49). Additionally, organoid models have been used to investigate adoptive cell immunotherapy and immune reconstitution, demonstrating their potential to advance cancer treatment approaches (50). In summary, organoid adoptive cellular immunotherapy is a potentially useful way to address the difficulties involved in converting preclinical research into viable cancer treatments.

### Immune checkpoint inhibitor response

A group of molecules known as immune checkpoint molecules controls the immune system and suppresses immune cells. They are crucial for immune system induction, self-tolerance maintenance, and the prevention of autoimmune disorders. Nevertheless, malignant tissues can use these spots to bypass the immune system, meaning that immune checkpoint protein molecules are ineffective in assisting the body’s defense against cancer. ICIs, often referred to as immune system counterpoint inhibitors, are medications that inhibit the immune system with the dual goals of increasing a patient’s immunity against malignancies and facilitating the immune system’s ability to escape from them.

Research has indicated that coculturing immune cells and cancer organoids can improve our understanding of immune checkpoint blockade treatments. For example, coculturing immune cells treated with bispecific anti-PD-1/PD-L1 antibodies with high-grade serous ovarian cancer organoids showed improved efficacy compared with coculturing immune cells treated with monospecific antibodies (51). However, because each patient has a unique resistance mechanism, not every patient responds to this medication. Research has concentrated on understanding the molecular correlates and genetic context of the immune checkpoint inhibitor response in various cancer types. A study conducted on Merkel cell carcinoma (MCC) sought to determine clinical genomic indicators linked to the immune checkpoint inhibitor response as well as to define the molecular landscape (52). Furthermore, changes in PBRM1 have been confirmed to be a reliable indicator of the immune checkpoint inhibitor response in renal cell carcinoma (RCC) (53). One important element determining the response to ICIs and targeted therapy is the TME. To optimize checkpoint inhibitor-TKI combinations in RCC, an *ex vivo* 3D tumor organoid model has been used, which has shown differences in T-cell activation and tumor cell killing efficacy among patient samples (54). Additionally, research has investigated the use of cutting-edge strategies to strengthen the immune response to ICIs. To enhance anticancer immunity, for example, a transdermal administration of ICIs mediated by cold atmospheric plasma (CAP) and microneedles has been devised (55). Research has concentrated on identifying biomarkers and possible treatment targets to enhance the immune checkpoint inhibitor response, in addition to investigating the TME and innovative delivery strategies. By targeting sialoglycans that prevent immune cell activation, targeted glycan degradation has been suggested to enhance the anticancer immune response (56). Additionally, to surmount genetic heterogeneity and immune evasion that restrict the therapeutic response to ICIs, an organoid-based screen has been employed to find epigenetic inhibitors that enhance antigen presentation and amplify T-cell-mediated cytotoxicity in breast cancer (57). Overall, the creation of immune organoids as preclinical models has provided researchers with a platform to investigate the TME and direct precision oncology treatment approaches. Researchers intend to increase the effectiveness of ICIs and broaden their use in the treatment of cancer by comprehending the processes underlying resistance to ICIs and investigating cutting-edge tactics to boost the immune response (58).

### Precision medicine and immunotherapy response prediction

**Figure 5:**
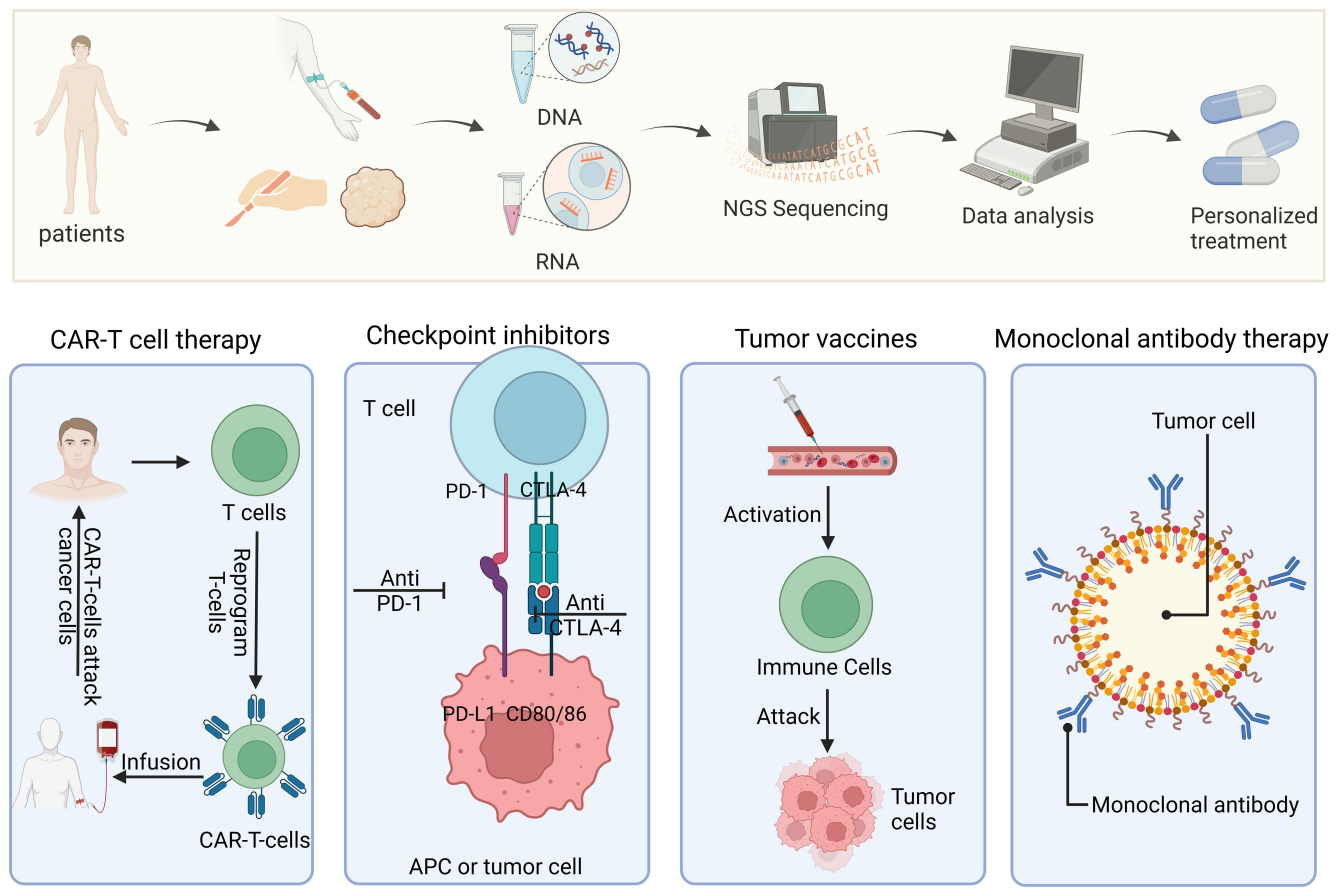
Precision medicine and personalized treatment process for immunotherapies. The diagram shows the entire process from patient sample collection and genome sequencing to personalized treatment plan formulation and implementation of different types of immunotherapies (such as CAR-T cell therapy, checkpoint inhibitors, tumor vaccines, and monoclonal antibody therapy).

Regarding immunotherapy response prediction, precision medicine is a rapidly developing discipline with enormous potential for tailored cancer treatment. The process of developing and implementing personalized immunotherapies is illustrated in **Figure 5**. Recent studies have highlighted the importance of biomarkers for predicting the efficacy of ICIs in cancer immunotherapy. One biomarker that has been proposed to be potentially significant in predicting the response to ICIs is the fecal microbiota (59). In individuals with recurrent or refractory classical Hodgkin lymphoma, circulating tumor DNA has also been found to be a predictive factor for therapeutic response (60).Finding predictive and prognostic biomarkers in the field of gastric cancer (GC) is now crucial for developing precision treatment plans (61). Given that GC is now understood to be a diverse disease, biomarkers are essential for determining which therapeutic strategy would work best for each patient.

Similarly, biomarkers have proven crucial in facilitating precise diagnosis and customized treatment regimens in allergic disorders and asthma (62). By offering insights into active tumor driving signal transduction pathways, developments in RNA-based diagnostic assays have also aided in the development of precision oncology techniques (63). These tests assist oncologists in selecting the most successful tailored therapy for patients by monitoring the activity of pertinent signaling pathways in tissue samples and circulating tumor cells. Using an *in vitro* system to culture numerous tumor tissues obtained from patients, Voabil et al. (64) introduced PD-1 antibodies to the culture system and measured the levels of four T-cell activation indicators, thirteen chemokines, and thirteen cytokines before and after adding the medication. The study revealed that the clinical responses of patients and cancer tissue response scores to therapy were quite similar, indicating that the model may be able to predict the early efficacy of PD-1 antibodies.

PDOs have become important instruments in precision oncology, enabling patients with hepatocellular carcinoma (HCC) to anticipate their response to treatment. Organoid-on-a-chip systems that are micro-engineered have been created to replicate the microenvironment of tumors and enable high-throughput drug screening for customized treatment approaches (65). The application of PDOs to predict patients’ individualized responses to conventional treatments is a hot research topic. Due to the difficulty in identifying biomarkers that can predict responses to immunotherapy, selecting the appropriate patient cohort to improve clinical response rates to ICIs remains challenging. However, comprehensive organoid culture systems, which include the TME, may have potential special roles.

In the patient-derived organotypic tumor spheroids (PDOTS) microfluidic culture containing tumor cells and autologous immune components, a one-week evaluation of T-cell-mediated tumor cell killing, and cytokine analysis showed that this model could predict or assess patients’ responses to ICI therapy (66). ALI organoids can also be used to assess T-cell function through flow cytometry, fluorescence staining, and tumor killing assays, thereby simulating responses to immune checkpoint inhibition. While the correlation of these predictive methods with clinical outcomes still needs to be validated, identifying cohorts with the best responses to immunotherapy through this platform provides substantial opportunities for clinical translation.

### Oncolytic virus therapy

A particular type of virus known as an oncolytic virus is capable of selectively infecting and killing cancer cells. They can propagate viruses to neighboring cells, obliterate tumor cells directly, and inhibit tumor-free host cells. This can stimulate the immune system and cause the release of cytokines (67). Oncolytic measles virus therapy has been shown by Packiriswamy et al. to boost T-cell responses against tumor-associated antigens in multiple myeloma patients (68). The use of oncolytic herpes simplex virus type 1 (HSV-1) for hematological malignancies was also investigated by Ishino et al. (69), who successfully killed a variety of cell lines generated from hematological malignancies. According to Elsedawy et al. (70), developments in oncolytic viral therapy have resulted in the creation of tailored picornaviruses that are designed as synthetic infectious RNAs. Furthermore, Bots et al. (71) investigated the use of adenoviruses derived from NHPs as possible oncolytic agents, emphasizing the significance of comprehending the distinctions and similarities between adenoviruses derived from NHPs and humans for prospective therapeutic applications.

Additionally, as Sugawara et al. (72) have shown, the combination of immune checkpoint inhibitors with oncolytic viral therapy has shown encouraging results in increasing antitumor responses. The use of a triple-mutated oncolytic herpes virus to treat cancers that grow quickly and slowly was also studied by Fukuhara et al. (73), emphasizing the significance of creating effective and safe oncolytic viruses for various tumor types. In general, enhancing the effectiveness of oncolytic virus therapy in treating different forms of cancer requires an understanding of the mechanisms by which these viruses modulate the immune response as well as the combination therapies they employ with ICIs. Raimondi et al. (74) established normal pancreatic organoids and pancreatic ductal adenocarcinoma (PDAC) tumor organoids to test the feasibility of using organoids as a screening platform for oncolytic adenovirus therapy. The results showed that the oncolytic adenovirus exhibited good selectivity, replicating only in PDAC organoids. This study also demonstrated the cytotoxicity of the oncolytic adenovirus and the individual variability in its synergistic effect with standard chemotherapy, indicating that organoids can serve as an appropriate model for preclinical responses to oncolytic virus therapy.

Moreover, in a study based on breast cancer (BC) organoids, researchers tested the efficacy of the measles vaccine virus and vaccinia virus for BC oncolytic therapy. The results suggested that all oncolytic viruses significantly inhibited the survival of BC organoids (75). The organoid platform can help in testing and designing oncolytic virus treatment regimens. However, the current research on oncolytic viruses mainly utilizes tumor organoid platforms. Tumor organoids have shown great potential in assessing the infectivity and cytotoxicity of oncolytic viruses, but there is a lack of experimental studies on the immune response elicited by oncolytic viruses. The application of immune organoids may bring new hope for oncolytic virotherapy.

## Therapeutic application of organoids in recent advanced treatment

Considered to be a breakthrough moment with great potential for customized medicine and cancer biology as a preclinical human tumor model, PDO technology has lately surged. Ma et al. (76) demonstrated the potential of organoids in the treatment of disease by creating an epithelial and mesenchymal organoid model obtained from human induced pluripotent stem cells for Localized Scleroderma (LoS) therapy. Similarly, Sahoo et al. (77) highlighted the potential of organoids to predict patient-specific responses to therapy by demonstrating the use of PDOs as an *ex vivo* model for the treatment of urothelial carcinoma (UC). Furthermore, Zhao et al. (78) demonstrated the promise of organoids in developing drug delivery systems for cancer treatment by proposing the use of nanotechnology and organoids to create cancer nanomedicines. Furthermore, the development of epidermal organoids (EpiOs) from iPSCs for skin regeneration was described by Kwak et al. (79), highlighting the therapeutic potential of extracellular vesicles (EVs) formed from organoids in regenerative medicine. Future developments in technology may lead to the widespread use of PDO models in cancer diagnosis and treatment, and more patients may gain from the use of the functional PDO assay in precision radiotherapy.

## Discussion

Organoids are emerging as a new technology in the field of antitumor immunology research, but they are still evolving. Immunocompetent cancer-on-chip (iCoC) systems with integrated immune components have surfaced in the last few years. The parameters of the sophisticated iCoC model TME may be precisely adjusted to more closely resemble the intricate relationships and dynamics that exist between immune system components and human tumor cells. The iCoC has been effectively applied in numerous immunotherapy studies. ICoC has been applied successfully in numerous immunotherapy studies (80).

Even while organoids show great promise for both clinical and basic cancer research, there are still a few difficult obstacles that need to be overcome. Initially, it is expensive to establish, maintain, and age organoids. Secondly, the organoids generated from the tissue samples are merely a tiny fraction of the whole tumor. Because tumors vary widely, it is unclear how reliable it is to use small portions of tumor tissue in place of the entire tumor. Translational cancer research can be reliably facilitated, and tumor heterogeneity better reflected by removing tissue from multiple regions of the same tumor. Thirdly, the complexity of immunological environments unique to each patient is difficult for present organoid technology to mimic. While co-culture systems containing immune and tumor-like cells have improved the modeling of tumor-immune interactions and their effect on therapy, several issues could prevent precise modeling and prediction of immunotherapy responses. For example, the immune components and cell counts of distinct tumor forms vary, which influences the immune cell composition in the early phases of tumor-like culture and the selection for preserving and growing these immune cells. While some tumor types only have immune cells in the surrounding stroma or do not have any immune cells at all, other tumor types do have numerous complicated types of immune cells.

There is no single model system that can perfectly replicate a tumor; instead, the tumor immune microenvironment is a complex, highly diverse, and dynamic system of numerous immune cells and stromal cells. Similar to the organoid/immune cell coculture model, there is a greater restriction on the kinds of immune cells that can be added externally. Additionally, the condition of the extraimmune cells varies greatly from that of the original TME and is not consistent with that of the original cells in the immune microenvironment. Additionally, immune cell survival and cellular activity may be hampered by the inability of organoid media to maintain immune cell function at its peak. On the other hand, in models of ALI or microfluidic culture, some of the initial immune cells that infiltrate the tumor remain within the organoid. These immune cell fractions are more likely to be lost during passaging or freeze-thawing and are only preserved for a brief period of time. As such, these techniques are limited to research that lasts a short time. More significantly, circulating immune cells interact intricately with tumor and tumor-infiltrating immune cells, and the tumor immunological microenvironment is constantly changing. These models do not account for circulating immune cells; instead, they consider only the local immunological profile of the TME. In immune organoid research, modeling susceptibility and resistance to immunotherapies—which include checkpoint inhibition and new pathways—represents a significant translational hurdle. Organoids can not only clarify possible resistance mechanisms but also support therapeutic endeavors such as drug screening and optimization of *in vitro* and cellular immunotherapies.

Multiple microenvironmental components could be integrated into the same system in future studies. Ultimately, the goal of the study should be to determine which modeling system is most appropriate, and the benefits and drawbacks of each system should be balanced. A greater technological investment should be made in TME components such as vascular and neural populations. Future improvements could be made to *in vitro* 3D models to improve tumor immunology research and clinical translation by more closely resembling the original tumor both physically and functionally.

### Summary and future perspectives

In addition to retaining the genomic and genetic characteristics, heterogeneity, passage stability, and the ability to be massively expanded *in vitro*, tumor organoids can also undergo gene editing without ethical issues. Therefore, this platform has significant advantages in evaluating combined immunotherapy strategies for cancer patients, screening new immunotherapy methods, and identifying biomarkers, making personalized precision treatment for patients possible.

Furthermore, tumor organoids derived from liquid biopsies are not limited by tumor location and can be dynamically observed at different stages of tumor progression or before and after treatment. Organoid technology enables the visualization of tumor growth processes.

Combining organoid culture technology with organ-on-a-chip technology allows for the integration of multiple components of the TME, aiming to study the intercomponent communication within this microfluidic system. This approach ensures controllable and reproducible organoid cultures and, to some extent, achieves organoid vascularization. Utilizing 4D imaging technology allows for the dynamic monitoring of live cell interactions between different cell subsets, which is particularly important for studying immunodynamics in co-culture systems.

Immune organoids have great potential in simulating the effects of immunotherapy, studying resistance mechanisms, and developing new combination treatment strategies. With continuous optimization of culture protocols, immune organoids are expected to drive the clinical translation of tumor immunotherapy, ultimately achieving personalized immunotherapy. Current and future organoid methodologies will greatly advance basic science and clinical research in immuno-oncology. It is anticipated that this approach will provide significant therapeutic benefits for patients, paving the way for promising breakthroughs in human tumor immunotherapy.

## Author contributions

YY: Writing – review & editing, Writing – original draft, Software, Resources, Project administration, Methodology, Formal analysis, Conceptualization. JC: Writing – review & editing, Visualization, Project administration, Investigation. YK: Writing – review & editing, Investigation, Conceptualization. YH: Writing – review & editing, Resources, Methodology. CM: Writing – review & editing, Software, Methodology, Investigation, Data curation.
